# The Safety of Laparoscopic Cholecystectomy in Super-elderly Patients: A Propensity Score Matching Analysis

**DOI:** 10.7759/cureus.42097

**Published:** 2023-07-18

**Authors:** Shigetoshi Naito, Masatoshi Kajiwara, Ryo Nakashima, Takahide Sasaki, Suguru Hasegawa

**Affiliations:** 1 Gastroenterological Surgery, Fukuoka University Hospital, Fukuoka, JPN

**Keywords:** population aging, a propensity score-matched analysis, super-elderly, laparoscopic, cholecystectomy

## Abstract

Background: Although reports on the safety of laparoscopic cholecystectomy (LC) exist, few have included patients aged ≥ 85 years. Hence, our study aimed to evaluate surgical outcomes of LC in patients aged ≥ 85 years.

Methods: After excluding patients who underwent other types of surgeries, 583 patients who underwent LC between 2015 and 2022 were included. Patients were classified into two groups based on age: < 85 years (control group, n = 551) and ≥ 85 years (super-elderly group, n = 32). Propensity score matching (PSM) was performed based on preoperative clinical parameters, and intraoperative and postoperative outcomes were compared.

Results: After PSM, 28 patients were included in each group. Intraoperative blood loss (1 vs. 5 mL, respectively; P = .052) and frequency of serious postoperative complications (Clavien-Dindo class ≥ 2, 2/28 (7.1%) vs. 6/28 (21.4%), P = .252) were similar between the control and elderly groups. There was no significant difference in the length of postoperative stay (control group: 5 (4-24) days vs. super-elderly group: 7 (3-64) days, P = .236). Unfortunately, one case of pneumonia of unknown cause occurred postoperatively, resulting in the death of one patient in the super-elderly group.

Conclusions: There were no clinically significant differences in the short-term outcomes of LC between super-elderly patients aged ≥ 85 years and patients aged < 85 years. Hence, LC may be relatively safe even in patients aged ≥ 85 years. However, owing to many pre-existing diseases and deterioration of physiological function, careful management during the perioperative period is desirable.

## Introduction

Population aging is a global phenomenon. The World Health Organization reported that the number of people aged ≥ 80 years increased to 143 million in 2019. Furthermore, this figure is projected to triple between 2019 and 2050, reaching 426 million [1]. Consequently, the number of surgeries for patients aged ≥ 80 years is expected to increase in the future. Compared with the general population, treatment methods for the elderly should be selected considering their general condition and activities of daily living due to age-related changes in cardiopulmonary and renal functions.

Regarding cholelithiasis, the prevalence of gallstones sharply increases with age. The prevalence of cholelithiasis has been reported to be 10% in men and 25% in women aged 60-69 years, whereas it increases to 24% in men and 35% in women aged ≥ 90 years [2]. Laparoscopic cholecystectomy (LC) is a safe, useful, and minimally invasive procedure for symptomatic cholecystitis [3,4]. In Japan, approximately 90% (32,178 cases) of all cholecystectomies were performed laparoscopically in 2017 [5]. Inevitably, an aging society will show an increase in the number of cholelithiasis cases. Therefore, in an aging society, the number of cases in which LC is performed, even in elderly people, will also increase.

Previous reports indicate that LC in the elderly has a high rate of intraoperative complications and is converted to open surgery [6-10]. However, recent studies have reported that LC outcomes in elderly patients are not significantly different from those of younger patients [11,12]. These reports were often segmented by the age of 80 years. Therefore, it is practical to study a more senior group, such as those aged ≥ 85 years.

Longer persistence of a gallstone is associated with a higher incidence of cancer [13,14]. In one study, biliary symptoms were significantly higher in the untreated group than in the cholecystectomy group, suggesting that cholecystectomy should be performed for bile duct stones associated with gallstones [15]. Thus, older adults with symptomatic gallstones may require surgery in the future. Surgical treatment is indicated for patients with preserved general conditions, including cardiopulmonary function, and indications for LC have similar requirements. However, the general condition of super-elderly patients is worse than that of young patients, making it difficult to determine the indications.

People in this age group tend to have a higher prevalence of pre-existing conditions and increased risks that are unavoidable. Additionally, there are potential risks associated with aging itself. Therefore, there may be significant concerns regarding the suitability of surgery for this age group. However, considering the future aging population, the possibility of performing LC on super-elderly individuals aged 85 and older is likely to increase.

However, the general condition of super-elderly patients is worse than that of young patients, making it difficult to determine the indications. Therefore, there may be significant concerns regarding the suitability of surgery for this age group. However, considering the future aging population, the possibility of performing LC on super-elderly individuals aged 85 and older is likely to increase. Hence, we conducted a comparison between the group aged 85 and older and the group below that age and additionally employed propensity score matching (PSM) analysis to equalize risks other than age and investigate whether age alone poses a risk.

## Materials and methods

After obtaining approval from the ethics committee, the surgical database of the institution was retrospectively searched for potentially relevant cases. Surgical indications included symptomatic cholelithiasis, chronic cholecystitis, acute cholecystitis, and preventive surgeries for the common bile duct stones and gallbladder polyps. Patients were divided into control and super-elderly groups according to their age at the time of surgery. PSM was performed based on preoperative clinical parameters. Patient characteristics, perioperative variables, and outcome variables were collected from the surgery database, hospital, and clinic charts.

The general condition of the patients was described according to the American Society of Anesthesiologists (ASA) classification. White blood cell count; hemoglobin levels; platelet levels; serum aspartate transaminase, alanine transaminase, albumin, blood urea nitrogen, and C-reactive protein levels; and prothrombin time-international normalized ratios were assessed. Creatinine clearance (CCr) was calculated using the Cockcroft-Gault formula as a marker of renal function. Postoperative complications were assessed and categorized according to the Clavien-Dindo classification (CDC) system. Data on survival and cause of death were also collected.

Perioperative management and surgical procedure

For elective surgery, we adhered to the policy of preoperative and intraoperative care, which states that cardiopulmonary function tests should be performed during elective surgery. In emergency surgery, percutaneous transhepatic gallbladder drainage or aspiration (PTGBD/A) is performed depending on the severity grade of cholecystitis and the propensity score of patients. LC was performed with a four-hole port, and the undulation was set to a pneumoperitoneum pressure of 10 mmHg with CO2. Although intraoperative cholangiography is not routinely performed, the cystic artery and duct were treated after dissecting the Calot’s triangle and confirming the critical view of safety. The surgical instruments used were dissection forceps, an L-shaped/spatula-type electric scalpel, and ultrasonic coagulation and incision devices. As a general rule, no drainage tube was placed; however, if intraoperative bleeding or damage to the gallbladder or gallbladder bed was observed, a closed drain was placed as required.

Ethical approval

This study was approved by the Ethics Committee of the Fukuoka University Hospital (U22-02-011). Due to the retrospective study design, informed consent was substituted for an informed opt-out procedure, and anonymized data were used.

Statistical analyses

Continuous variables were expressed as medians with ranges. The propensity score was calculated using a logistic regression model. The purpose of this PSM was to reduce confounding factors other than age that could be the risk of postoperative complications and to examine whether age itself could be a risk. Therefore, we selected sex, BMI, ASA score, emergency surgery, and PTGBD/A as possible preoperative risks. Matching was performed using a 1:1 matching protocol without replacement calipers with a width of 0.20 of the standard deviation of the log of the propensity score. Statistical comparisons were performed using the chi-square test or Fisher's exact test, and Student's t-test was used to analyze differences between continuous values. All P-values were two-sided, and P-values < .05 were considered statistically significant. All statistical analyses were performed using EZR (Saitama Medical Center, Jichi Medical University), a graphical user interface for R (The R Foundation for Statistical Computing, Vienna, Austria, version 2.13.0) [16].

## Results

Search results and study characteristics

**Figure 1:**
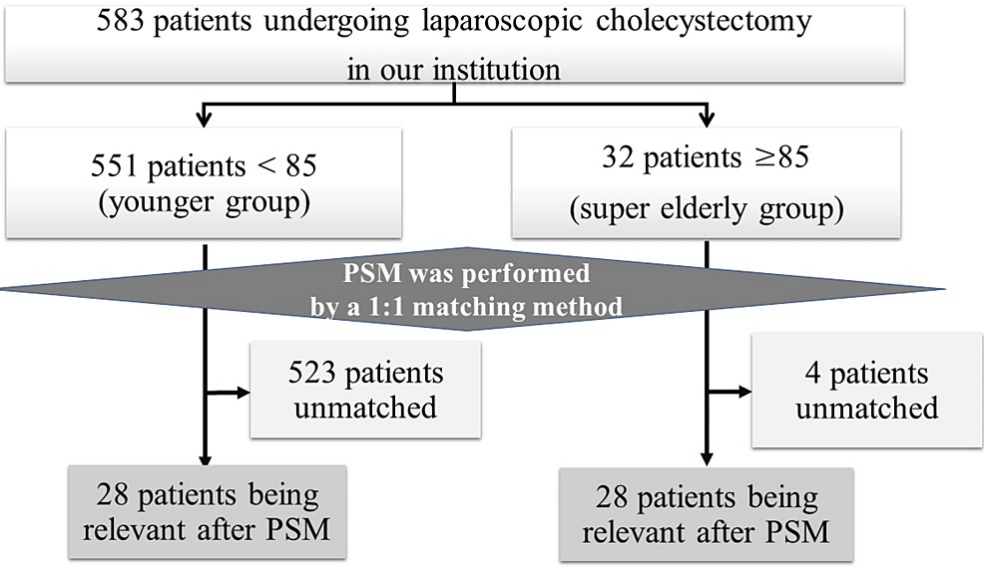
Flow chart outlining the sequential steps of the study PSM: propensity score matching

After excluding patients who underwent other types of surgery, 583 patients who underwent LC between 2015 and 2022 were included in the study. Figure 1 shows the diagram of the study. Accordingly, 32 patients aged ≥ 85 years comprised the super-elderly group, and 551 patients aged < 85 years comprised the control group. Each data point was examined before and after PSM.

Table 1 presents the patients’ characteristics. Table 2 shows significant differences between the pre-PSM control group and the super-elderly group: history of treatment for hypertension (27.9% vs. 57.1%, respectively; P < 0.01), history of treatment for heart failure (1.3% vs. 12.5%, respectively; P < 0.01), history of treatment for coronary artery disease (8.5% vs. 25.1%, respectively; P < 0.01), and history of ischemic heart disease (1.1% vs. 9.4%, respectively; P = 0.01). Table 3 shows no difference in inflammatory values and clotting cysts; however, CCr was poor (76.5 vs. 59.7 mL/min, respectively; P < 0.01) in the control and super-elderly groups. The ratio of ASA class ≥ 3 was higher in the super-elderly group than that in the control group.

**Table 1:** Background characteristics of patients in the cohort BMI: body mass index, PSM: propensity score matching

Variables	Before PSM			After PSM		
	Control group (n= 551)	Super-elderly group (n= 32)	P-value	Control group (n= 28)	Super-elderly group (n= 28)	P-value
Age, y, median (range)	66 (14-84)	86 (85-93)	<0.01	67 (50-84)	86 (85-93)	<0.01
Sex, n (%), female	261 (47.4)	11 (34.4)	0.20	8 (28.6)	8 (28.6)	1
Male	290 (52.6)	21 (65.6)		20 (71.4)	20 (71.4)	
BMI (range)	23.48 (14.59-39.09)	23.92 (16.97-33.73)	0.62	23.92 (16.95-33.87)	23.92 (16.97-33.73)	0.97

**Table 2:** Concurrent diseases of patients in the cohort PSM: propensity score matching, ASA: American Society of Anesthesiologists, TIA: transient ischemic attack, NA: not available

Variables	Before PSM			After PSM		
	Control group (n= 551)	Super-elderly group (n= 32)	P-value	Control group (n= 28)	Super-elderly group (n= 28)	P-value
ASA, n (%), 0-2	482 (87.5)	18 (56.2)	<0.01	18 (64.3)	18 (64.3)	1
3 or higher	69 (12.5)	14 (43.8)		10 (35.7)	10 (35.7)	
Hypertension	154 (27.9)	17 (53.1)	0.04	13 (46.4)	16 (57.1)	0.59
Diabetes mellitus	72 (13.1)	5 (15.6)	0.60	9 (32.1)	4 (14.3)	0.21
History of congestive heart failure	7 (1.3)	4 (12.5)	0.01	1 (3.6)	3 (10.7)	0.61
History of ischemic heart disease	47 (8.5)	9 (28.1)	0.01	10 (35.7)	6 (21.4)	0.38
History of treatment for valvular disease	6 (1.1)	3 (9.4)	0.01	1 (3.6)	2 (7.1)	1
History of cerebral infarction/TIA	36 (6.5)	4 (12.5)	0.27	5 (17.9)	4 (14.3)	1
History of upper abdominal surgery	16 (2.9)	1 (3.1)	1	1 (3.6)	1 (3.6)	1
Current hemo-/peritoneal dialysis	4 (0.7)	0 (0.0)	1	28 (100.0)	28 (100.0)	NA
Mental disorder	26 (4.7)	3 (9.4)	0.21	0 (0.0)	3 (10.7)	0.24

**Table 3:** Preoperative laboratory data of patients in the cohort PSM: propensity score matching, WBC: white blood cell, PT-INR: prothrombin time-international normalized ratio, AST: aspartate transaminase, ALT: alanine transaminase, BUN: blood urea nitrogen, CCr: creatinine clearance, CRP: C-reactive protein

Variables	Before PSM			After PSM		
	Control group (n= 551)	Super-elderly group (n= 32)	P-value	Control group (n= 28)	Super-elderly group (n= 28)	P-value
WBC (×103/μL;range)	6.3 (2.6-8.2)	6.2 (3.0-9.4)	0.82	6.55 (3.8-17.0)	5.8 (3.0-15.3)	0.33
Hgb (g/dL;range)	13.0 (7.4-13.1)	12.9 (7.8-17.3)	0.59	13.1 (10.3-17.2)	12.8 (7.8-17.3)	0.18
Plt (×104/μL;range)	21.80 (5.4-67.9)	22.4 (9.0-47.5)	0.72	22.5 (21.8-40.9)	19.7 (9.0-40.1)	0.31
PT-INR	1.03 (0.83-2.76)	1.06 (0.95-1.61)	0.43	1.02 (0.91-1.14)	1.09 (0.95-1.61)	0.16
AST (IU/dl;range)	22 (4-911)	24 (13-74)	0.28	22 (13-61)	25 (13-74)	0.16
ALT (IU/dl;range)	20 (3-794)	22 (5-82)	0.56	20 (3-74)	22 (5-82)	0.54
Alb (mg/dl;range)	3.9 (2.2-5.9)	3.8 (2.4-4.7)	0.37	3.9 (3.2-5.5)	3.75 (2.4-4.7)	0.10
BUN (mg/dl;range)	14 (4-73)	13 (5-26)	0.54	14.5 (5-21)	13.0 (5.0-26.0)	0.46
Cr (mg/dl;range)	0.77 (0.38-15.07)	0.80 (0.41-4.42)	0.43	0.84 (0.50-1.34)	0.81 (0.41-4.42)	0.53
CCr※ (ml/min;range)	76.5 (3.2-236.3)	59.7 (10.7-103.1)	<0.01	72.2 (36.5-131.1)	57.3 (10.7-103.1)	0.01
CRP (mg/dl;range)	0.15 (0.10-38.96)	0.16 (0.10-26.38)	0.74	0.21 (0.10-25.70)	0.12 (0.10-26.38)	0.38

Perioperative characteristics and postoperative morbidity

Table 4 shows the perioperative factors related to the operative procedures and postoperative morbidity. There was no difference in the incidence of acute cholecystitis and PTGBA/D; however, the bleeding volume was significantly higher in the super-elderly group than that in the control group. Before PSM, the length of hospital stay after surgery was longer in the super-elderly group compared to the control group. However, the median surgical blood loss difference was between 1 g and 5 g or median length of hospital stay after surgery was between five and seven days, and the differences were not statistically significant. In contrast, no difference was observed in the rate of conversion to laparotomy (control group: 1.1% vs. super-elderly group: 3.1%; P = 0.33) and the incidence of clinically problematic CDC grade ≥ 2 (control group: 7.6% vs. super-elderly group: 15.6%; P = 0.17).

**Table 4:** Factors concerning operative procedures and postoperative morbidity PSM: propensity score matching, PTGBD/A: percutaneous transhepatic gallbladder drainage or aspiration, CDC: Clavien-Dindo classification

Variables		Before PSM			After PSM		
		Control group (n= 551)	Super-elderly group (n= 32)	P-value	Control group (n= 28)	Super-elderly group (n= 28)	P-value
Acute cholecystitis (%)		184 (33.4)	15 (46.9)	0.13	12 (42.9)	13 (46.4)	1
Performed PTGBD/A (%)		57 (10.3)	5 (15.6)	0.41	7 (25.0)	7 (25.0)	1
Urgent operation (%)		73 (13.2)	3 (9.4)	0.79	2 (7.1)	2 (7.1)	1
Operation time, min, median (range)		142 (22-479)	134 (52-261)	0.77	142 (43-479)	184 (51-371)	0.04
Surgical blood loss, g, median (range)		1 (1-900)	5 (1-679)	0.01	1 (1-227)	5 (1-679)	0.05
Conversion to laparotomy, n (%)		6 (1.1)	1 (3.1)	0.33	1 (3.6)	1 (3.6)	1
Postoperative complication, n (%)							
CDC	0	497 (90.2)	25 (78.1)	0.07	26 (92.9)	20 (71.4)	0.29
	1	12 (2.2)	2 (6.2)		0 (0.0)	2 (7.1)	
	2	27 (4.9)	3 (9.4)		1 (3.6)	3 (10.7)	
	3	12 (2.2)	1 (3.1)		1 (3.6)	2 (7.1)	
	4	1 (0.2)	0 (0.0)		0 (0.0)	0 (0.0)	
	5	2 (0.4)	1 (3.1)		0 (0.0)	1 (3.6)	
CDC≧2 (%)		42 (7.6)	5 (15.6)	0.17			
Bile leakage (%)		4 (0.7)	1 (3.1)	0.25	1 (3.6)	1 (3.6)	1
Intra-abdominal abcess (%)		3 (0.5)	0 (0.0)	1			
Common bile duct stone (%)		11 (2.0)	1 (3.1)	0.50	1 (3.6)	1 (3.6)	
Surgical site infection (%)		2 (0.4)	0 (0.0)	1			
Pneumonia (%)		5 (0.9)	2 (6.2)	0.05	0 (0.0)	2 (7.1)	0.49
The falling accident (%)		1 (0.2)	0 (0.0)	1			
Cardiovascular disease (%)		4 (0.7)	0 (0.0)	1			
Bowel inflammation (%)		1 (0.2)	0 (0.0)	1			
Delirium (%)		1 (0.2)	0 (0.0)	1			
Multiple organ failure (%)		2 (0.4)	0 (0.0)	1			
Bacteremia (%)		0 (0.0)	1 (3.1)	0.06	0 (0.0)	1 (3.6)	1
Others		5 (0.9)	1 (3.1)	0.29	0 (0.0)	1 (3.6)	1
Operative mortality, n (%)		2 (0.4)	1 (3.1)	0.16	0 (0.0)	1 (3.6)	1
Length of postoperative stay, d, median (range)		5 (1-74)	7 (3-64)	0.01	5 (4-24)	7 (3-64)	0.24

Table 4 details the complications that were CDC grade ≥ 2. Although there was a higher rate of heart disease preoperatively, there was no difference in cardiovascular conditions. Unfortunately, pneumonia of unknown cause occurred postoperatively, and one case of death occurred in the super-elderly group.

PSM analysis

Adjustments were made for sex, BMI, ASA score, emergency surgery, and PTGBA/D for PSM. Ultimately, 28 patients each were included in the super-elderly and control groups in the PSM analysis (Figure 1). After PSM, the differences in the preoperative items disappeared, except for CCr. In addition, the statistical difference before PSM in terms of intraoperative bleeding and postoperative hospital stay disappeared. However, the operation time in the super-elderly group was longer than that in the control group (142 vs. 184 min, respectively; P = 0.04).

## Discussion

In this study, we investigated the short-term post-LC outcomes of super-elderly patients (≥ 85 years) compared with those of the younger population. We also investigated postoperative complications in detail. No clinically significant differences were observed in the short-term outcomes of LC between super-elderly patients aged ≥ 85 years and those aged < 85 years.

In this study, super-elderly patients had poorer ASA scores than younger patients because of cardiovascular disease. The relationship between ASA scores and postoperative complications is well known [17], and a difference in the postoperative complication rate was expected in this study. However, no difference was observed in the conversion to laparotomy, and the incidence of complications of grade ≥ 2 CDC included biliary tract complications. In some studies, the complication rates were significantly higher in patients aged > 80 years [6,18]. This complication rate may be related to the surgical rate for acute cholecystitis. There is a difference in the degree of difficulty of surgery in challenging cases of cholecystectomy, such as acute cholecystitis, fibrotic gallbladder, and Mirizzi syndrome, resulting in complications [6,19]. This study included not only acute cholecystitis but also various other diseases. However, there was no difference in the ratio of acute cholecystitis to emergency surgery, and we considered these biases to be low.

If older age is an independent risk factor for complications, we would expect to see differences even in the absence of differences in ASA scores. We found no difference in the rate of ASA score ≥ 3 after PSM and no difference in perioperative outcomes. Hence, even if the ASA score is poor but well managed, LC can be considered safe for super-elderly people. However, the operation time of the super-elderly group was longer than that of the control group. No significant differences in surgical blood loss were observed between the control and super-elderly groups; however, the amount of surgical blood loss was greater in the super-elderly group, and the tissue fragility of the elderly and as typified by senile purpura may have required hemostasis, affecting the total operation time. Pneumonia was the most frequent complication in the super-elderly group. There was one case of death due to pneumonia of unknown cause. Some studies have reported that age is an independent risk factor for postoperative pulmonary complications [20]. Previous reports have indicated that pneumonia is a major complication of LC in elderly patients (≥ 65 years) [21]. Therefore, it is important to pay close attention to potential complications specific to elderly adults.

This study has some limitations. First, this was a single-center retrospective observational study. Several variables, such as biological factors, may have introduced bias into the analysis. Second, experienced surgeons may opt for laparoscopy when choosing an approach even in older patients. Third, the investigation of cholecystitis severity assessed by the Tokyo Guideline 2018 was retrospective, and data collection was challenging. Early LC in cases of acute cholecystitis and complex disease is associated with less surgical stress and shorter hospital stays [4]. Potential biases, such as the selection of alternative treatments for more severe cases, cannot be ruled out. Therefore, prospective control trials should be conducted in the future.

## Conclusions

No clinically significant differences were observed in the short-term outcomes of LC between super-elderly patients aged ≥ 85 years and younger patients. Hence, LC may be relatively safe even in patients aged ≥ 85 years. However, owing to pre-existing diseases and deterioration of physiological function, careful management during the perioperative period is desirable. Given the small sample size in this study, further research is required to verify the study findings.
